# *In situ* self-assembly of gold nanoparticles on hydrophilic and hydrophobic substrates for influenza virus-sensing platform

**DOI:** 10.1038/srep44495

**Published:** 2017-03-14

**Authors:** Syed Rahin Ahmed, Jeonghyo Kim, Van Tan Tran, Tetsuro Suzuki, Suresh Neethirajan, Jaebeom Lee, Enoch Y. Park

**Affiliations:** 1 Research Institute of Green Science and Technology, Shizuoka University, 836 Ohya Suruga-ku, Shizuoka 422-8529, Japan; 2 Department of Cogno-Mechatronics Engineering, Pusan National University, Busan 609-735, Korea; 3 Department of Infectious Diseases, Hamamatsu University School of Medicine, 1-20-1 Higashi-ku, Handa-yama, Hamamatsu 431-3192, Japan; 4 School of Engineering, University of Guelph, Guelph, ON N1G 2W1, Ontario, Canada; 5 Graduate School of Science and Technology, Shizuoka University, 836 Ohya Suruga-ku, Shizuoka 422-8529, Japan

## Abstract

Nanomaterials without chemical linkers or physical interactions that reside on a two-dimensional surface are attractive because of their electronic, optical and catalytic properties. An *in situ* method has been developed to fabricate gold nanoparticle (Au NP) films on different substrates, regardless of whether they are hydrophilic or hydrophobic surfaces, including glass, 96-well polystyrene plates, and polydimethylsiloxane (PDMS). A mixture of sodium formate (HCOONa) and chloroauric acid (HAuCl_4_) solution was used to prepare Au NP films at room temperature. An experimental study of the mechanism revealed that film formation is dependent on surface wettability and inter particle attraction. The as-fabricated Au NP films were further applied to the colorimetric detection of influenza virus. The response to the commercial target, New Caledonia/H1N1/1999 influenza virus, was linear in the range from 10 pg/ml to 10 μg/ml and limit of detection was 50.5 pg/ml. In the presence of clinically isolated influenza A virus (H3N2), the optical density of developed color was dependent on the virus concentration (10–50,000 PFU/ml). The limit of detection of this study was 24.3 PFU/ml, a limit 116 times lower than that of conventional ELISA (2824.3 PFU/ml). The sensitivity was also 500 times greater than that of commercial immunochromatography kits.

Numerous promising approaches to fabricating nanostructured films have been developed to enhance their surface and interfacial properties by achieving an appropriate combination of surface structure, morphology, and physical and chemical properties. Such nanostructured films are increasingly used in a broad range of applications, including energy storage^1^,^2^,^3^,^4^,^5^,^6^,^7^, electronics^8^,^9^,^10^,^11^,^12^,^13^,^14^,^15^, and biomedicine^16^,^17^. In particular, noble-metal NP films have been extensively studied because of their versatility, chemical stability, and unique optical properties stemming from localized surface plasmon resonances, which lead to enormous local, nanoscale optical field enhancements. This phenomenon finds applications in optical devices, microelectromechanical devices, sensors, medical devices, and catalysis^18^,^19^.

Among plasmonic materials, gold nanoparticles (Au NPs) are one of the most promising materials because of their unprecedented ability to confine electromagnetic fields and potential applications in a vast array of photonic and optoelectronic technologies ranging from chemical sensing and biosensing applications to energy harvesting^4^,^20^,^21^, imaging^22^, data storage^23^, and optical tweezer devices^24^ for nanomaterial manipulation. Because of these possible applications of Au NP films, many conventional and Nano technological methods have been proposed for film fabrication, including electron-beam lithography and nanoimprint lithography as top-down techniques^25^,^26^. However, these methods require sophisticated fabrication equipment and their applications are limited by either high costs or small sample sizes. Alternatively, wet-chemical approaches have advantages over lithographically fabricated films because wet-chemical building blocks enable the fabrication of highly crystalline materials on a large scale. Furthermore, simple bottom-up approaches based on self-assembly, such as Langmuir-Blodgett film deposition, dip coating, and electrochemical deposition, have also been developed for large-scale fabrication to obtain films with much fewer defects; however, these techniques are time-consuming and laborious^27^,^28^,^29^,^30^.

Zhang *et al*. reported a novel and simple *in situ* fabrication technique to prepare Au NP-PDMS free-standing films that do not require additional reducing agents; these films can be used in enzyme reactors, immunoassays, and other biochemical analyses on microchips^27^. Goyal *et al*. have also reported a simple one-step method for synthesizing noble-metal NP-embedded free-standing PDMS composite films. Their process involves preparing a homogenous mixture of the metal salt (Ag, Au, or Pt), a silicone elastomer, and a curing agent (hardener) to form NPs when the hardener crosslinks with the elastomer^31^. However, such techniques are limited to PDMS substrates. Therefore, two major limitations must still be overcome to advance the industrial applications of plasmonic Au NP films. Among the more urgent challenges in the nanobiotechnological field is the development of a method for preparing Au NP plasmonic films on different substrates, regardless of the substrates’ hydrophilic or hydrophobic character. Such films would play an important role in the development of next-generation flexible electronic devices, display devices, hybrid nanostructures, surface-enhanced Raman scattering (SERS) substrates and immunoassays. The need to develop facile *in situ* self-assembling Au NP films on a large scale is another urgent challenge to reduce the time and labor involved and the amount of waste generated, as well as to push plasmonic films toward practical applications.

In this article, we present a simple approach to fabricate Au NP films *in situ* on various types of substrates. Our fabrication process is a one-step procedure that involves a mixture of sodium formate and an HAuCl_4_ solution and can be used to obtain Au NP films on any hydrophilic or hydrophobic substrate, e.g., glass, 96-well polystyrene plates or PDMS. SERS and immunosensing applications were demonstrated films fabricated via the proposed method. The Au NP films were further applied to develop a colorimetric sensor to detect influenza virus A as a model analyte because of the high demand for sensitive and accurate diagnosis to prevent infection at early stages^1^.

## Results

### Preparation of Au NP films on different hydrophilic and hydrophobic substrates

**Figure 1:**
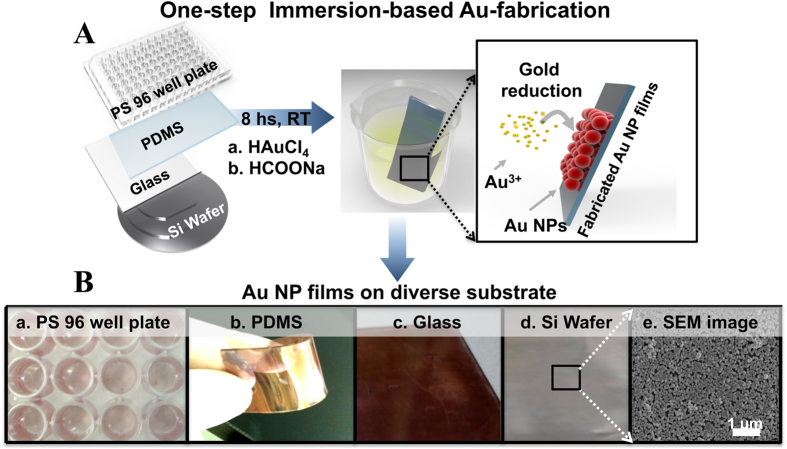
Presentation of the single-step, immersion-based fabrication of Au NP films on diverse substrates. (**A**) Procedure for the fabrication of the Au NP films. (**B**) Photographic images of Au NP films deposited on 96-well polystyrene plate (a), PDMS (b), glass (c), silicon wafers (d) and SEM image of densely packed Au NPs on silicon substrate (e).

In the first step, the method for the *in situ* self-assembly of the Au NPs was applied to different substrates to determine the method’s versatility, i.e., 96-well PS plate, PDMS substrate, glass and silicon wafer (Fig. 1A). The films turned a reddish color, indicating the good deposition of the particles. UV-Vis spectroscopy was used to monitor the surface plasmon absorption of the Au NPs. As the concentration of HCOONa (reducing and stabilizing agent) was increased, the peak absorption centered at 548 nm (at 200 mM HCOONa) was slightly blue shifted, indicating that the particle size decreased (Fig. S1, Supplementary Information). However, a second peak correlated with the NPs and abnormal shapes were not obtained in this concentration range (Figs S1 and S2, Supplementary Information). The film height and size of the NPs were 30 and 40 nm, respectively. The SEM image showed good deposition of the NPs on the silicon wafer substrate (Fig. 1B-e). This deposition method represents a wet chemical approach for the facile, one-step, self-assembling fabrication of highly packed plasmonic films at room temperature using different substrates.

**Figure 2:**
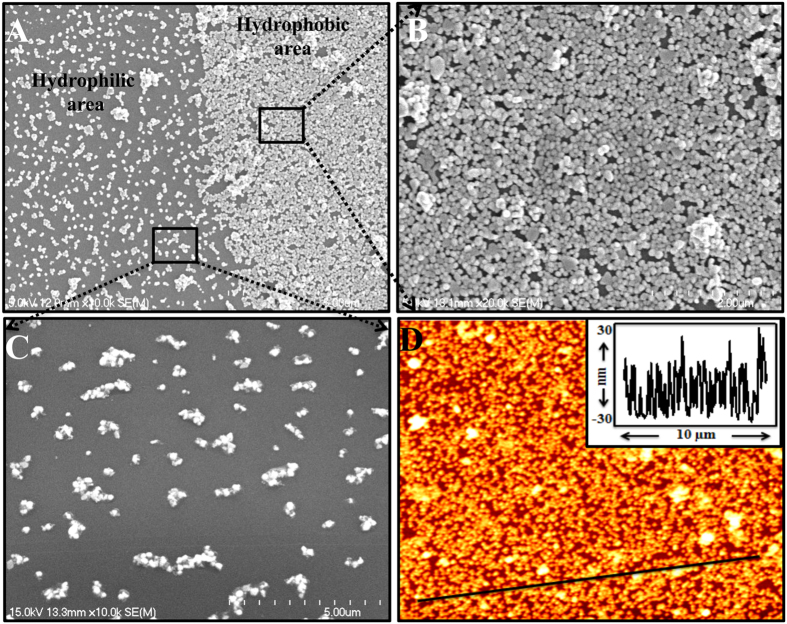
Microscopic study of the films. (**A**–**C**) SEM images of Au nanoparticles (NPs) deposited onto the surface of unmodified and modified PDMS substrates. (**D**) AFM image of Au NPs deposited onto the hydrophobic PDMS substrate (the inset shows the depth profile along the line).

In addition, the Au NPs were deposited onto hydrophilic and hydrophobic PDMS substrates, where part of the hydrophobic PDMS sample was treated with O_2_ plasma (Fig. S3, Supplementary Information). The SEM images of both surfaces revealed that Au NP films formed on both surfaces. However, the Au NPs were more densely deposited onto the hydrophobic surface than onto the hydrophilic surface (Fig. 2A–C). Figure 2D shows an AFM image of the Au NP film deposited onto hydrophobic PDMS surface. The average height of the film was approximately 30 nm. With this method, all the NPs were involved in the film formation and the remaining bulk solution was colorless. The absorbance spectra of the bulk solution and film showed the absence of plasmonic peaks in the remaining solution after the Au NPs were deposited onto the hydrophobic surface (Fig. S4).

### Mechanistic study of Au NP deposition onto the substrates

**Figure 3:**
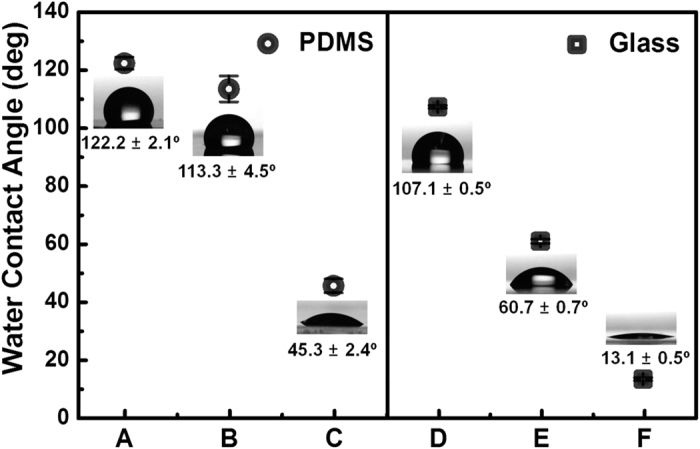
Mechanistic study of Au NP deposition onto a substrate. Static water contact angle (*θ*_st_) values were measured on PDMS and glass with different treatments. Insets: digital images of water droplets (10 μL in volume) placed onto each substrate.

To investigate the mechanism by which the Au NPs were deposited onto the substrates, we prepared PDMS and glass substrates treated under different conditions, which resulted in different surface wettabilities (Table S1 and Fig. 3). The static contact angle (*θ*_st_) of the as-prepared PDMS (sample B) significantly decreased from 113.3 ± 4.5° to 45.3 ± 2.4° after oxygen plasma treatment (sample C). The patterned PDMS surface (sample A) with a regular microstructure displayed a slightly increased surface hydrophobicity (*θ*_st_ = 122.2 ± 2.1°). Untreated glass (sample E) appeared to be relatively hydrophobic (*θ*_st_ = 60.7 ± 0.7°) and became highly hydrophilic (*θ*_st_ = 13.1 ± 0.5°) after piranha washing (sample F). Hydrophobic glass (sample D, *θ*_st_ = 107.1 ± 0.5°) was obtained by self-assembly of OTS on the piranha-cleaned surface.

The morphology of the Au films deposited onto the substrates was observed using SEM (Fig. S5). In the PDMS substrates, a remarkable difference was observed in the Au film deposited onto the as-prepared and plasma-treated PDMS surfaces. The density of the Au NPs on the plasma-treated PDMS was much lower than that of the as-prepared PDMS. However, no difference was observed between the as-prepared and patterned PDMS surfaces. A similar phenomenon was observed for the glass substrates, where the density of Au NPs on the hydrophobic (OTS treated) and relatively hydrophobic (untreated) surfaces was much higher than that on the hydrophilic surface (piranha treated).

In the particle-substrate system, the interaction forces can be classified into long-range (e.g., an der Waals, electrostatic and hydrophobic interactions) and short-range (metallic, ionic, covalent and hydrogen bonds) forces^32^,^33^. The adhesion of nanoparticles onto substrate surfaces has been attributed primarily to van der Waals, electrostatic, hydrophobic and capillary adhesion forces^34^,^35^,^36^. The results for both the PDMS and glass substrates show that Au NPs preferentially adhere to a hydrophobic surface rather than to a hydrophilic one. Because of the relative surface hydrophobicity of the uncoated Au NPs (*θ*_st_ ≈ 70°)^37^,^38^, the strong adhesion of the Au NPs to the hydrophobic surface is most likely governed by hydrophobic interactions, as demonstrated in many previous studies^39^,^40^,^41^. At the hydrophobic surface, water molecules in contact with hydrophobic moieties tend to be displaced into the aqueous solution to reduce the total free energy of the system, resulting in a favorable interaction between the hydrophobic moieties of substrate surfaces and the Au NPs. The hydrophobic interaction is suppressed when the substrate surface is hydrophilic. Additionally, because of the electrokinetically negative surface of bare Au^42^, the highly negatively charged surface of clean glass and oxygen-plasma-treated PDMS induce electrostatic repulsion toward the NPs, resulting in a sparsely deposited Au film. The electrostatic interaction is partially responsible for the different morphologies of the Au films on the hydrophobic PDMS and hydrophobic (relative hydrophobic) glass surfaces (Fig. S5A,B and S5D,E). As shown in Fig. S5B and D, the hydrophobicity of the two different surfaces is similar (107.1° and 113.3°), but the deposited Au films have reasonably different coverages of NPs, which is attributed to the different surface potentials of PDMS and glass. The cleaned and OTS-modified glass surfaces are highly negatively charged (−48.64 ± 0.07 and −35.15 ± 0.36 mV, respectively)^41^. The untreated glass surface is also negatively charged, but the charge is weaker than that of the cleaned glass. The as-prepared and patterned PDMS surfaces are neutrally charged^43^. The Au NPs also form large aggregates on the glass (untreated, cleaned and OTS-treated) and oxygen-plasma-treated surfaces. However, the Au NPs are fairly dispersed on the hydrophobic PDMS surfaces. This dispersion is attributed to the hydrodynamic interactions between the particles and substrate, which induce long-range attractive forces between the identically charged surfaces of the Au NPs and the substrate^44^.

### Observation of surface-enhanced Raman scattering effect on Au NP films

**Figure 4:**
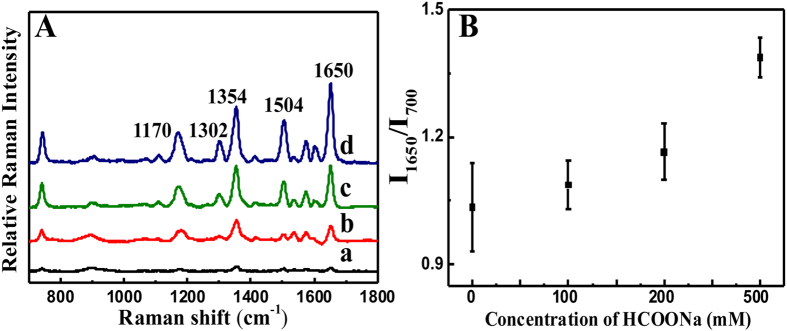
Surface-enhanced Raman scattering of Au NP films deposited onto the PDMS substrate. (**A**) SERS profiles of Au nanoparticle films deposited onto PDMS surfaces with (a) 0 mM, (b) 100 mM, (c) 200 mM and (d) 500 mM HCOONa. (**B**) The calibration curve of the Raman intensity versus HCOONa concentration. Error bars in (**B**) denote standard deviation (n = 3).

Figure 4 shows the SERS profiles of the R6G molecules on the Au NP films that were formed using different concentrations of HCOONa. The SERS enhancement of the Au NP films increased with increasing HCOONa concentration. The strongest SERS enhancement was achieved from the film fabricated with 500 mM HCOONa (Fig. 4A). The integrated intensity of the R6G 1650 cm^−1^ Raman band from the Au film prepared using 500 mM HCOONa was four times stronger than that of the films prepared using100 mM HCOONa (Fig. 4B). The SERS enhancements observed in the nanostructured Au films (obtained with 500 mM HCOONa) resulted from the deposition of many Au NPs on the surface, increasing the film surface roughness, which promotes electromagnetic field enhancements, resulting in more active sites for molecule adsorption.

### Metal-enhanced fluorescence (MEF) study of the Au NP films

**Figure 5:**
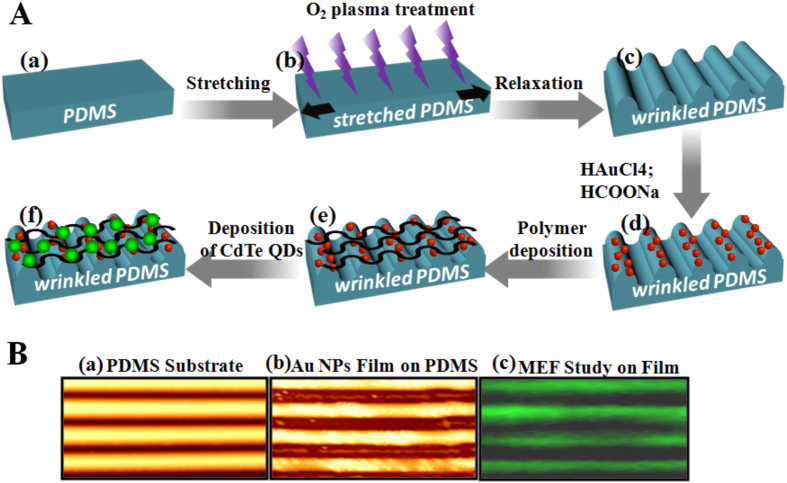
Metal-enhanced fluorescence study on Au NP film. (**A**) Schematic presentation of wrinkled PDMS substrate preparation (a–c), Au NP film fabrication on it (d) and CdTe QDs deposition using layer-by-layer method (e,f). (**B**) AFM image of bare wrinkled PDMS substrate (a), Au NP film formation on wrinkled PDMS substrate (b) and fluorescence microscopy image of Au NP film/QDs hybrid structure (c).

To evaluate the optical properties of the Au NP films, CdTe QDs were synthesized and immobilized on Au NPs deposited on wrinkled PDMS substrates using our previously reported method (Fig. 5A)^1^. The absorbance and PL intensity of QDs were shown in Fig. S6A. The absorbance shoulder of QDs is located at 502 nm, while the PL peak of QDs is situated at 522 nm. The particles size of QDs was estimated about 2.85 nm and its concentration was 2.17 × 10^−6^ M. A total internal reflection fluorescence microscope (Leica AM TIRF MC, Germany) was used to study metal-enhanced fluorescence on the Au NP films. AFM image of pattered PDMS substrate shown in Fig. 5B-a where pale yellow lines and reddish lines indicate tip positions and down parts, respectively. Au NPs film formation was confirmed with color change of PDMS substrate (reddish), and also AFM images showed some white spotted (Au NPs) on film (Fig. 5B-b). In such patterned film structure, it was very hard to deposit Au NPs spontaneously on down part of the substrate due to narrow gap between two tip position (2 μm) (Fig. S7). Therefore, a huge amount of Au NPs deposited on tip position of PDMS substrates in compare to down position. Figure 5B-c shows a fluorescence microscopic image of the QD/polymer-deposited Au films on PDMS pattern; strong PL enhancement induced by metal-enhanced fluorescence was observed from tip position of substrate because the absorption and/or emission bands of the QDs overlap with the plasmonic scattering wavelength of the Au NP films.

### Confirmation of anti-HA antibody immobilization on Au NPs films

Before bioassay experiment, the presence of adlayer formic acid (HCOOH) on film and its stability in water and under UV light were examined. As shown in Fig. S8, FTIR peaks at 1350 cm^−1^, 1625 cm^−1^ and 3000–25000 cm^−1^ represent the presence of symmetric OCO stretch, asymmetric OCO stretch and carboxylic group O-H stretch, respectively, on Au NPs films. Zeta potential value of Au NPs film was measured at −36.5 eV. In addition, adlayer functional group showed strong stability under UV light irradiation (red line in Fig. S8) and water (black line in Fig. S8) with no change of characteristic functional group in FTIR spectra. To illustrate the feasibility of using Au nanostructured films for bioassay applications, antibodies against influenza virus HA were conjugated with the Au NP films deposited onto a 96-well polystyrene plate. The ELISA results confirmed the successful conjugation of the anti-influenza A virus HA H1 antibodies (HA Ab 66189, Abcam, Cambridge, UK) with adlayer carboxylic group (COO^−^) on Au NP films. As shown in Fig. S9, both the layer-by-layer and EDC/NHS chemistry methods resulted in a higher optical density of the anti-HA antibody-conjugated Au NP films than that of the unmodified Au NP films, suggesting that the nanostructured film was successfully conjugated to the antibodies.

### Immunoassay for virus detection

**Figure 6:**
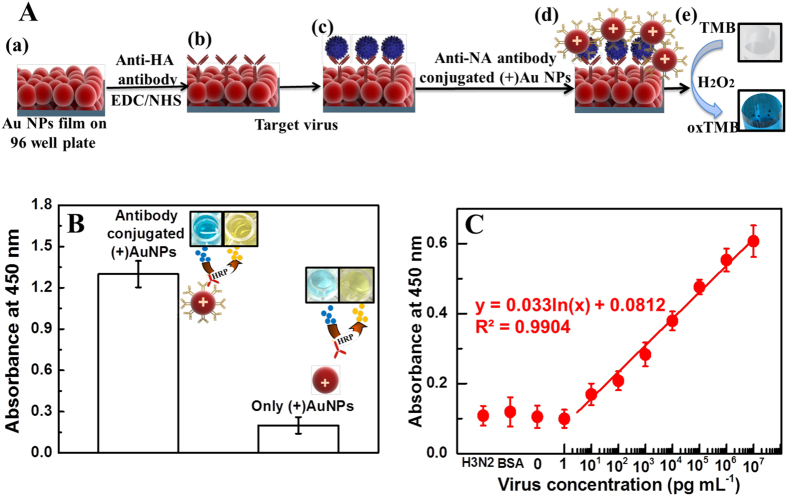
Detection of influenza virus A (New Caledonia/20/1999) (H1N1) on films. (**A**) Schematic of virus detection: (a) Au NP films on a 96-well polystyrene plate; (b) anti-HA antibody immobilization on Au NPs through EDC/NHS chemistry. (c) After the NPs were washed three times, the target virus was added and incubated for 30 min at room temperature. (d) The anti-NA antibody-conjugated (+)Au NPs bound the virus through an antibody-antigen reaction, and the unbound (+)Au NPs were washed out. (e) TMB-H_2_O_2_ was added, and rapid color changes were observed because of the oxidation of peroxidase substrate TMB (oxTMB). (**B**) ELISA results of anti-NA antibody binding to the (+)Au NPs through electrostatic interactions. (**C**) The calibration curve of the absorbance corresponding to the concentration of the influenza virus A (New Caledonia/20/1999) (H1N1). BSA was used as a negative control; H3N2 donates influenza virus A/Yokohama/110/2009 (H3N2) that was used to check specificity of the system. Error bars in (**B**) and (**C**) denote standard deviation (n = 3).

In this study, a sensitive and quantitative method to detect influenza virus A (H1N1) was developed; this method is based on the peroxidase-like catalytic activity of Au NP films and colloidal (+)Au NPs toward TMB-H_2_O_2_ mixtures (Fig. 6A). The absorbance spectra of the synthesized (+)Au NPs were shown in Fig. S6B. The absorbance peak of the (+)Au NP solution was located at 526 nm and its concentration was 3.5 × 10^−10^ M. The surface charge of (+)Au NPs was + 34.5 mV (Zetasizer, Nano-ZS, Malvern, UK). Furthermore, transmission electron microscope revealed (+)Au NP with an average size of 40 nm (Fig. S6C). The conjugation of the anti-NA antibody with (+)Au NPs was confirmed using ELISA (Fig. 6B). Then, biofunctionalized Au NP films and (+)Au NPs formed a hybrid structure with different concentrations of influenza virus A (New Caledonia/20/1999) (H1N1) (where the surface of this virus has specific binding sites for the anti-HA Ab and anti-NA Ab), and catalytic activity was monitored. In the quantitative analysis using different concentrations of the target virus, the absorbance logarithmically corresponded to the virus concentration in the range of 10 pg/ml to 10 μg/ml (Fig. 6C) and limit of detection was calculated as 50.5 pg/ml (Based on standard deviation method)^45^. However, no substantial color developed in the case of BSA and the H3N2 virus.

**Figure 7:**
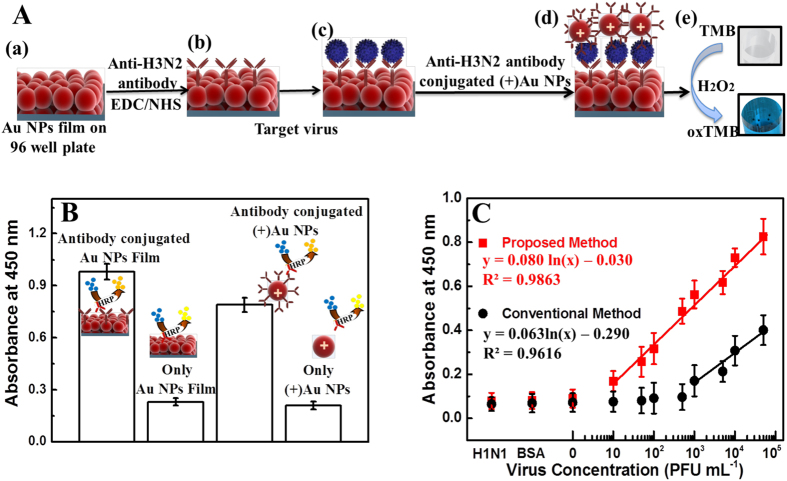
Detection of influenza virus A/Yokohama/110/2009 (H3N2) on films. (**A**) Schematic of virus detection: (a) Au NP films on a 96-well polystyrene plate; (b) anti-H3N2 antibody immobilization on Au NPs through EDC/NHS chemistry. (c) After the NPs were washed three times, the target virus was added and incubated for 30 min at room temperature. (d) The anti-H3N2 antibody-conjugated (+)Au NPs bound the virus through an antibody-antigen reaction, and the unbound (+)Au NPs were washed out. (e) TMB-H_2_O_2_ was added, and rapid color changes were monitored. (**B**) ELISA results of binding confirmation of anti-H3N2 antibody binding to the Au NP film and (+)Au NPs. (C) The calibration curve of the absorbance corresponding to the concentration of the influenza virus A/Yokohama/110/2009 (H3N2). BSA was used as a negative control; H1N1 donotes influenza virus A (New Caledonia/20/1999) (H1N1) that was used to check specificity of the system. Squares (red line) and circles (black like) denote proposed and conventional ELISA sensing results, respectively. Error bars in (**B**) and (**C**) denote standard deviation (n = 3).

Using this developed monitoring system, an influenza virus A/Yokohama/110/2009 (H3N2) was monitored (Fig. 7A). The specificity of anti-H3N2 HA MAb for influenza virus A/Yokohama/110/2009 was confirmed in our previous study^45^. The binding of anti-H3N2 HA MAb with Au NPs film and (+)Au NPs was also confirmed using ELISA (Fig. 7B). Then, the sensitivity of influenza virus A/Yokohama/110/2009 (H3N2) detection was observed in the range of 10–10,000 plaque forming units (PFU)/ml (Fig. 7C). The detection limit was shown at ca. 24.3 PFU/ml.

The sensitivity of detection in this study was compared with that of a commercially available rapid influenza diagnostic kit, which we have studied previously^1^ and the conventional ELISA method on Au NPs film (Table 1 and Fig. 7C). A linear response to virus detection with ELISA was seen up to 1000 PFU/ml with limit of detection 2824.3 PFU/ml, whereas in the ImunoAce Flu kit, the linear response was seen up to 5000 PFU/ml. This indicated that our system is 116 times and 500 times more sensitive than conventional ELISA and commercial immunochromatography, respectively.

The versatility of this proposed sensing method was also tested on glass substrate fabricated with Au NPs film using avian influenza virus (A/Avian/Vietnam 1203/04, H5N1). After confirmed successful conjugation of anti-HA antibodies with Au NPs film and anti-NA antibodies with (+) Au NPs (Fig. S10A), different concentration of recombinant avian influenza virus was mixed with those bio-functionalised nanomaterials. A linear response up to 10 pg/ml with LOD value of 4.5 pg/ml was successfully observed and proved the versatility of proposed sensing method (Fig. S10B). A naked eye photographic images of sensing results on glass slide were shown in Fig. S11. Overall, this new strategy of *in situ* plasmonic film fabrication can be used to develop simple, highly sensitive and low-cost colorimetric point-of-care diagnosis techniques for infectious viral detection without any complex equipment or trained operator.

## Discussion

With advancements in processing technologies and a wide range of applications, the science of nanostructured films has garnered much interest and has become a flourishing field. Here, we have reported a wet-chemical-based, single-step, *in situ* procedure for fabricating Au NP films on different substrates at room temperature. The Au NPs were well distributed on both hydrophilic and hydrophobic surfaces. Here, we limited our fabrication to a centimeter length area; however, the procedure is readily scalable. The limitation of the scalability of plasmonic fabrication on different substrates (including rigid and flexible substrates) can be overcome by this approach. Prospective applications of the proposed method include plasmonic light harvesting at low cost, stretchable electronic devices and plasmonic arrays for biomedical applications. The Au NP films were further applied to the development of a sensitive assay that can detect influenza virus A (H1N1) and A (H3N2) at concentrations of 10 pg/ml and 10 PFU/ml respectively. The sensitivity of this study was improved about 116 times and 500 times than conventional ELISA and commercial immunochromatography, respectively. The versatility of this proposed sensing method was also successfully examined on glass substrate using three kinds of influenza viruses. Therefore, we believe that the approach reported here opens new possibilities for a wide range of practical applications.

## Methods

### Materials

Gold (III) chloride trihydrate (HAuCl_4_·3H_2_O) was purchased from Sigma-Aldrich (St. Louis, MO, USA). Sodium formate (HCOONa) and sodium tetrahydroborate (NaBH_4_) were purchased from Wako Pure Chemical Inc. (Osaka, Japan). 1-Ethyl-3-(3-dimethylaminopropyl) carbodiimide (EDC), *N*-hydroxysuccinimide (NHS), poly-l-lysine (PLL), poly-diallyldimethylammonium chloride (PDDA; M.W. 400,000–500,000), poly-acrylic acid (PAA; M.W., ~450,000) and Rhodamine 6 G were purchased from Sigma-Aldrich (Milwaukee, USA). The anti-influenza A (H1N1) virus hemagglutinin (HA) antibody [B219M] (ab661189, lot: GR40088-11), anti-influenza A (H5N1) virus hemagglutinin (HA) antibody [2B7] (ab135382, lot: GR100708-16) and recombinant influenza virus A (Avian/Vietnam/1203/04) (H5N1) (lot: GR301823-1) were purchased from Abcam, Inc. (Cambridge, UK). The anti-neuraminidase (NA) (New Caledonia/20/1999/(H1N1) rabbit polyclonal antibody was obtained from Immune Technology Corp. (New York, NY, USA). Recombinant influenza virus A (New Caledonia/20/1999) (H1N1) (Cat No. 11683-V08H) and influenza A (H3N2) hemagglutinin monoclonal antibodies (Anti-H3N2 antibody HA MAb, Lot: HB04N0160) were purchased from Sino Biological, Inc. (Beijing, China). Avian influenza H5N1 neuraminidase polyclonal antibody (Cat No. PA5-34949) was purchased from Thermo Fisher Scientific (Rockford, USA). Multitest glass slide was purchased from MP Biomedicals, LLC (Eschwege, Germany). Dr. C Kawakami of the Yokohama City Institute of Health, Japan, kindly provided clinically isolated influenza virus A/Yokohama/110/2009 (H3N2). The chromogenic substrate, 3,3′,5,5′-tetramethylbenzidine (TMB) was obtained from Dojindo (Osaka, Japan). The ECL^TM^ anti-mouse IgG, horseradish peroxidase (HRP)-conjugated whole antibody was purchased from GE Healthcare UK Ltd. (Buckinghamshire, UK). All experiments were performed using highly pure deionized (DI) water (>18 MΩ
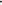
cm).

### Coating of Au NPs onto different substrates

A solution for Au NP coating was prepared where the ratio between the metal precursor and reducing agent was 1:5; i.e., a 100–500 mM aqueous solution of HCOONa (4 ml) and a 20 mM aqueous solution of HAuCl_4_ (1 ml) were added to 36 ml of DI water at room temperature (Fig. 1). Here, HCOONa used to reduce Au ions and stabilized Au NPs; hence, it’s a modified of Turkevich Method^46^. The substrate to be deposited with the Au NPs was then placed in the solution in a sloping position for 8 h at room temperature. The substrates were washed several times with DI water to remove the loosely bound nanoparticles. The film color turned reddish, indicating that the particles were deposited.

### Preparation of the hydrophilic and hydrophobic substrates

The different substrates with either hydrophilic or hydrophobic surfaces were prepared as follows:

1. Creating surfaces with different wettabilities on the PDMS substrate (Sample A, B and C in Table S1): To create a highly hydrophobic PDMS surface, micropillar arrays were fabricated using the conventional casting method (sample A). For this, approximately 60-μm-thick Si micropillars were first formed on a silicon substrate using a photolithography process. The PDMS precursor polymer was then mixed with a curing agent at a weight ratio of 10:1, poured onto the Si mold, and degassed in a vacuum desiccator for 1 h to remove the air bubbles. The substrate was subsequently subjected to thermal curing at 70 °C for 1 h; after it solidified, the PDMS substrate was carefully removed. The substrate was then briefly rinsed with DI water and blown dry with a nitrogen stream. Sample B is a natural PDMS elastomer surface without any treatment. The mixture of PDMS prepolymer and curing agent was poured into a leveled Petri dish and thermally cured at 70 °C for 1 h. Cured PDMS was washed with DI water and blown dry with a nitrogen stream. The hydrophobic surface of PDMS was made hydrophilic through oxygen plasma treatment (O_2_ pressure: 0.2 mbar, 99 W power) for 3 min (Sample C).
2. Creating surfaces with different wettabilities on a glass substrate (D, E and F in Table S1): Conventional micro cover glasses (18 × 18 mm^2^ and 0.12–0.17 mm thick, Matsunami Glass Ind., Osaka, Japan) were used to study the mechanism of Au NP fabrication. The cover glasses were sonicated in acetone for 30 min and then washed with DI water; this procedure was repeated 3 times. The washed glass substrate was used as Sample E. The cleaned glasses were then immersed in piranha solution for 30 min. Piranha-treated glass has a characteristic hydrophilic surface and was used as Sample F. To create a hydrophobic glass substrate, *n*-octadecyltrichlorosilane (OTS) was self-assembled onto the glass surface (Sample D). The piranha-treated glass was dipped in a 1 mM OTS solution (dissolved in toluene) for 1 h. The OTS-assembled glass was then sonicated in toluene for 1 min and washed with ethanol and water.

The different substrates with either hydrophilic or hydrophobic surfaces were immersed vertically in HAuCl_4_ and HCOONa mixture solution for overnight. The surface wetting properties were characterized through measurement of the static contact angle (SCA) using a contact-angle meter (DSA 20E, Krüss, Hamburg, Germany) equipped with a CCD camera module. The SCAs were measured with a 10 μl DI water droplet using a sessile drop method.

### Observation of the surface-enhanced Raman scattering (SERS) effect on the Au NP films

The nanostructured Au films obtained with solutions containing various concentrations of HCOONa were subjected to SERS experiments with Rhodamine 6 G (R6G) as the test molecule. The commercially pure reagents were dissolved in pure water (18.2 MΩ
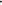
cm) and methanol, and the Raman scattering of the R6G/Au NP films was observed using a Raman spectroscope (Ramboss 500i, Dongwoo Optron, Kwangju, Korea).

### Preparation of wrinkled PDMS template for metal enhanced fluorescence (MEF) study

The wrinkled PDMS templates were fabricated based on previous report with a slight modification^47^. Briefly, prepared PDMS strips (10 × 30 × 5 mm) were fixed in custom-built stretching stages, and stretched by strain (ε) of 15%, then treated with oxygen plasma for 10 min (O_2_ pressure: 0.2 mbar, 99 W power). A thin glass-like layer was formed on the surface of the PDMS during the plasma treatment process which was released after strain. Here, the mechanical mismatch between the solidified skin layer and soft body layer causes buckling instabilities. Finally, a microscopically well-defined 1-dimensional surface wrinkle was arisen. Then, a mixture solution of HCOONa (500 mM) and HAuCl_4_ (20 mM) were added to wrinkled PDMS substrates at room temperature for 1 h. The substrates were washed several times with DI water to remove the loosely bound nanoparticles. The surface of PDMS substrates were acquired a negative charge at this stage due to the carboxylic group covered nanoparticles deposition. Then, the negatively charged substrates were used for polyelectrolyte deposition beginning with PDDA by means of layer-by-layer assembly method. Briefly, 1% PAA and 1% PDDA were prepared in aqueous media. The substrates were immersed in PAA or PDDA solution for 10 min, followed by washing in DI water for 10 min in order to assemble two layers of PDDA and PAA. The final stage was immersion in PDDA to confer strong positive electrostatic charges. Then, the substrates were washed with DI water and dried under ultrapure N_2_ gas. This sample was immersed in synthesized thioglycolic acid (TGA)-capped cadmium telluride (CdTe) quantum dots (QDs) solution^48^ for 10 min, at this time the final concentration of the QD solutions was adjusted to the nanomolar level to prevent aggregation with DI water. The polymer spacer layer between nanocrystals and metal surface avoids unwanted quenching effects due to radioactive energy transfer from nanocrystals to metal surface and assists in photo-luminescence (PL) enhancement^1^.

### Immobilization of the anti-influenza A virus hemagglutinin (HA) antibody and anti-H3N2 antibody HA MAb on the Au NP films

To check the stability of the film and its applicability in immunoassays and other bioassays, anti-HA influenza A virus antibody (HA ab 66189) was immobilized on the Au NP films using two well-known methods, i.e., the layer-by-layer method and EDC/NHS chemistry. Negatively charged carboxylic groups were present on the surface of the Au NP film in 96-well polystyrene plates. In the layer-by-layer method, after the film was rinsed several times with DI water, positively charged PLL (0.1% wt., Sigma-Aldrich) was electrostatically bound to the film, which was subsequently used to physically immobilize the anti-HA antibody on the surface of the nanostructured film. For EDC-NHS chemistry, 100 μl of EDC (4 mM) and 100 μl of NHS (10 mM) were added to all the wells of the nanostructured 96-well polystyrene plates, which were incubated for 10 min. Then, 1 μl of the HA ab66189 antibody (final concentration = 5 ng/ml) was added to each well and incubated at 4 °C for 24 h. The plate was washed three times with a phosphate-buffered saline (PBS) solution using an immunowasher (Bio-Rad, Model 1575, Segrate, Italy) to remove the nonspecific or unbound components. The antibodies bind to the Au film either through the amide bond or simply by electrostatic interaction. To determine whether the antibodies bound to the film, the samples were blocked with 100 μl of 2% bovine serum albumin for 2 h at room temperature. Anti-mouse immunoglobulin G (IgG)-horseradish peroxidase (HRP) (1 ng/ml, Santa Cruz Biotechnology, Santa Cruz, CA) was added to each sample. After incubation at room temperature for 1 h, the samples were washed three times with PBS solution. HRP was developed with 100 μl of the TMB substrate solution (10 μg/ml TMB, 10% H_2_O_2_ in 100 mM NaOAc, pH = 6.0) for 5–30 min at 25 °C. A blue-colored solution was obtained at this stage. The reaction was halted by the addition of 100 μl of 10% H_2_SO_4_. The solution then turned yellow, and its absorbance was measured at 450 nm with a reference at 655 nm using a microplate reader (model 680, Bio-Rad, Hercules, CA, USA). To conjugate anti-H3N2 antibody HA MAb with film, same procedure with EDC/NHS chemistry as described above was followed in this study.

### Synthesis of positively charged gold nanoparticles ((+)Au NPs)

The (+)Au NPs were prepared according to a published protocol^45^. Briefly, a cysteamine solution (400 μl, 213 mM) was added to 40 ml of a 1.42 mM HAuCl_4_ solution. After the resulting solution was stirred for 20 min at 25 °C, 10 μl of 10 mM NaBH_4_ solution was added and the mixture was vigorously stirred for 10 min at 25 °C in the dark. The mixture was then further stirred for 15 min, and the resulting wine-red solution was stored at 4 °C until further use.

### Conjugation of (+)Au NPs with anti-influenza A virus neuraminidase (NA) antibodies and anti-H3N2 antibody HA MAb

After the supernatant was separated by ultracentrifugation (Kubota 6200, Tokyo, Japan) at 5,000 rpm for 30 min, the (+)Au NPs were dispersed in Milli-Q water. Then, 1 ml of the synthesized (+)Au NP solution and 1 μl of anti-NA antibody (New Caledonia/20/1999/(H1N1) (final concentration of 5 ng per ml) were incubated for 30 min and maintained at 4 °C for 24 h. At this stage, the NPs were conjugated with the antibody through electrostatic interactions. The conjugated (+)Au NP-antibodies were separated from their unconjugated or nonspecific binding partners through centrifugation (5,000 rpm for 30 min) and redispersed in 1 ml of Milli-Q water. To confirm that the antibody had bound to the (+)Au NPs, the samples were blocked with 100 μl of 2% BSA for 2 h at 25 °C. One ng per ml anti-rabbit IgG-HRP (Santa Cruz Biotechnology) was added to each sample. After incubation at 25 °C for 1 h, the samples were washed 3 times with PBS solution. HRP was developed with 100 μl of TMB substrate solution (10 μg per ml TMB, 10% H_2_O_2_ in 100 mM NaOAc, pH 6.0) for 5–30 min at 25 °C. A blue solution developed at this stage. The reaction was stopped by the addition of 100 μl of 10% H_2_SO_4_. The solution then became yellow in color, and its absorbance was read at 450 nm using a microplate reader (model 680, Bio-Rad, Hercules, CA). Same procedure was followed to conjugate and binding confirmation of (+) Au NPs with anti-H3N2 antibody.

### Detection of influenza virus A/New Caledonia/20/1999(H1N1) and A/Yokohama/110/2009 (H3N2)

The anti-HA Ab (ab66189)-conjugated Au NP films were rinsed 3 times with PBS. Then, 100 μl of anti-NA Ab-conjugated (+)Au NPs containing different concentrations of recombinant influenza A (H1N1) were added to the microplate wells. An Ab-conjugated (+)Au NP solution in BSA and influenza virus A (H3N2) were added to the same microplate as a negative control. The microplate was incubated for 30 min at room temperature. After the microplate was washed three times, 100 μl of a TMB and H_2_O_2_ (5 nM) solution was added to each of the wells. The microplate was then incubated for 1 min at 25 °C. Finally, 100 μl of 10% H_2_SO_4_ was added to each of the wells as a stopping reagent. The absorbance of the color was read at 655 nm using a plate reader (Model 680, Bio-Rad, Hercules, CA). A dose-response curve was constructed on the basis of the absorbance values using different concentrations of influenza virus A (New Caledonia/20/1999) (H1N1). Here, the proposed sensing assay may offer a new strategy to monitor influenza virus with high sensitivity through the conjugation of different antibodies to the film and nanoparticles. The same protocol as described above was used to detect influenza virus A/Yokohama/110/2009 (H3N2) using anti-HA (H3N2) Ab-bioconjugated Au NPs film and (+)Au NPs.

### Spectroscopy and structural characterization

The ultraviolet-visible (UV-Vis) spectra of the nanostructured films were recorded using a Tecan Infinite M 200 spectrophotometer (Infinite^®^ F500, TECAN Ltd., Männedorf, Switzerland). Topographic images of the Au NPs films were obtained using atomic force microscopy (AFM, diInnova, Veeco, USA) and scanning electron microscopy (SEM, S4700, Hitachi High-Technologies Co., Minato-ku, Japan).

## Additional Information

**How to cite this article:** Rahin Ahmed, S. *et al*. *In situ* self-assembly of gold nanoparticles on hydrophilic and hydrophobic substrates for influenza virus-sensing platform. *Sci. Rep.* **7**, 44495; doi: 10.1038/srep44495 (2017).

**Publisher's note:** Springer Nature remains neutral with regard to jurisdictional claims in published maps and institutional affiliations.

## Supplementary Material

Supplementary Information

## Figures and Tables

**Table 1:** Comparison of influenza virus A/Yokohama/110/2009 (H3N2) detection using different methods.

Detection method	Virus concentration (PFU/mL)								
	10000	5000	1000	500	100	50	10	1	0
This Study	+	+	+	+	+	+	+	−	−
Conventional ELISA	+	+	+	−	−	−	−	−	−
Commercial kit^1^	+	+	−	−	−	−	−	−	−

*Note: + and − denote the positive and negative diagnoses, respectively.
