# Supplementary material for: In situ self-assembly of gold nanoparticles on hydrophilic and hydrophobic substrates for influenza virus-sensing platform
Source: Sci Rep. 2017 Mar 14;7:44495. doi: 10.1038/srep44495 (PMC5349514; doi:10.1038/srep44495)
Supplement: Supplementary Information [file srep44495-s1.pdf]

Supplementary information

In situ self-assembly of gold nanoparticles on hydrophilic and hydrophobic substrates for influenza virus-sensing platform

Syed Rahin Ahmed[a‡], Jeonghyo Kim[b‡], Van Tan Tran[b], Tetsuro Suzuki[c], Suresh Neethirajan[d], Jaebeom Lee[b,*], and Enoch Y. Park[a,e,*]

E-mail:
rahin_sust@yahoo.com (SRA)
yaamako@gmail.com (JK)
trantan160288@gmail.com (VTT)
tesuzuki@hama-med.ac.jp (TS)
sneethir@uoguelph.ca (SN)
jaebeom@pusan.ac.kr (JL)
park.enoch@shizuoka.ac.jp (EYP)

[*]Corresponding authors. *E-mail address:* park.enoch@shizuoka.ac.jp (EYP), jaebeom@pusan.ac.kr (JL)

[a] *Research Institute of Green Science and Technology, Shizuoka University, 836 Ohya Suruga-ku, Shizuoka 422-8529, Japan*

[b] *Department of Cogno-Mechatronics Engineering, Pusan National University, Busan 609-735, Korea*

[c] *Department of Infectious Diseases, Hamamatsu University School of Medicine, 1-20-1 Higashi-ku, Handa-yama, Hamamatsu 431-3192, Japan*

[d]*School of Engineering, University of Guelph, Guelph, ON N1G 2W1, Ontario, Canada*

[e]*Graduate School of Science and Technology, Shizuoka University, 836 Ohya Suruga-ku, Shizuoka 422-8529, Japan*

[‡]Both contributed equally.

Table S1: Substrates preparation with different conditions.

			A	B	C
PDMS	Treatment		Micro patterned surface	Natural PDMS surface	O_2 Plasma treatment (99 W, Air gas 9, 3 min)
	$HAuCl_4$ (Precusor)		1 mL, 20 mM		
	HCOONa (Reducing agent)	Positive	4 mL, 500 mM		
		Negative	4 mL, 0 mM		
	Reaction time		Overnight dipping, 8 hrs		
			D	E	F
Glass slide	Treatment		Assembly of OTS*	Natural cover glass surface	Piranha cleaning
	$HAuCl_4$ (Precusor)		1 mL, 20 mM		
	HCOONa (Reducing agent)	Positive	4 mL, 500 mM		
		Negative	4 mL, 0 mM		
	Reaction time		Overnight dipping, 8 h		

* n-Octadecyltrichlorosilane

Deposition of Au NP Films on polystyrene 96 well plates and glass substrate

Au NP film was generated in the surface of the polystyrene 96 well plate since this plate has been widely utilized in biological experiment. The addition of Au surface on the plate might apply to immunoassay using gold binding proteins or Au-thiol bioconjugation, as well as fluorescence enhancement. Figure S1 shows the UV-vis spectra of Au NPs formed on the polystyrene 96 well plates. In this case, 50 μL of HCOONa (200 nm) and $HAuCl_4$ (20 mM) mixture solution was added in each of the wells at room temperature for overnight.

When 100 mM of sodium formate as reducing agent was added to grow the NPs, there was no unique color change in the substrate. Indeed, the UV-vis spectrum showed no plasmonic peak. (Fig. S1a). When the substrate was immersed in the $HAuCl_4$ and HCOONa solution mixture from 200 mM, the film became red gradually, indicating the formation of Au NPs.

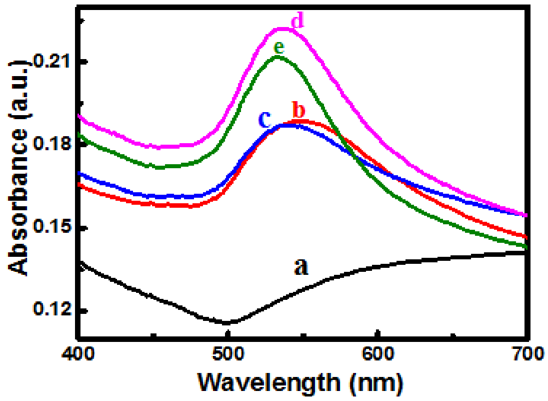

Figure S1. Ultraviolet-visible absorption spectroscopy of Au nanoparticle (NP) films deposited on polystyrene 96 well plates with different concentrations of HCOONa: (a) 100 mM; (b) 200 mM; (c) 300 mM; (d) 400 mM and (e) 500 mM.

Then, the glass substrate (micro cover glass, 18×18 mm and 0.12–0.17 mm thick, Matsunami Glass Ind., Osaka, Japan) was adopted to be coated with the same process. Figure S2(A) shows their UV-vis spectra depending on the reducing agent. As similar to the spectra of Au film on the polystyrene plate, the optical spectra showed NP constructing when 100 mM of sodium formate was added to the solution. Figure S2(B)show the surface-view SEM images of nanostructured layers formed on glass with different HCOONa concentrations. It is clearly seen that with increase in the HCOONa concentration, the NPs overlap with each other and form densely packed films. Use of 100 mM HCOONa fails to form nanostructured films (Fig. S2B-a). However, with increase in the HCOONa concentration, Au NPs were formed and deposited on the substrate (Fig. S2B-b, c, and d).

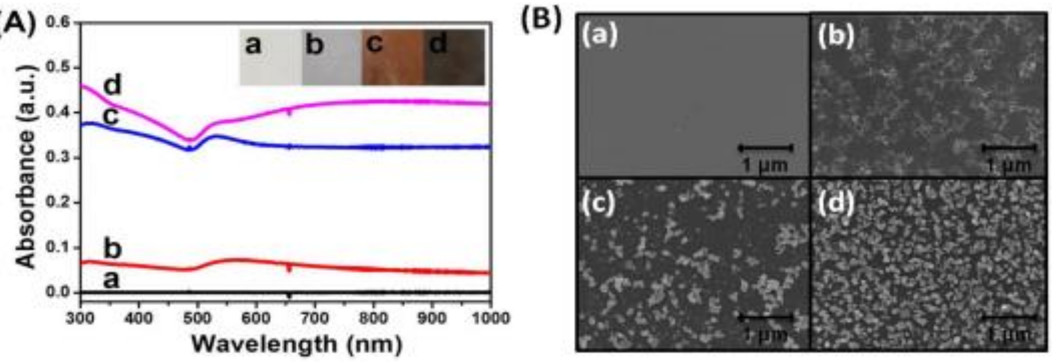

Figure S2. (A) UV-Vis absorption spectra and photographs of the Au NPs fabricated glass substrate using different concentration of HCOONa; a (0 mM), b (100 mM), c (200 mM), d (500 mM). Insets show the color of the glass substrates after deposition. (B) Scanning electron microscope images of nanostructured films on glass, which were deposited with solutions containing (a) 0 mM, (b) 100 mM, (c) 200 mM, and (d) 500 mM HCOONa.

Preparation of PDMS substrate and its surface modification

PDMS substrates were prepared based on previously reported.[1] A mixture of precursor polymer and hardener at a ratio of 10:1 was used to prepare PDMS elastomer, then degassing and casting into a leveled petri dish. After gelation at room temperature for one day, the PDMS was cured at 80°C for 5 h. Stripes with a width of 1 cm and a length of 4 cm were cut out of the resulting 2 mm thick elastomer plate. To remove dust, the stripes were shortly rinsed with deionized water and blown dry in a nitrogen stream. Subsequently, the stripes were fixed in custom-built stretching stages, strained by varying degrees, and treated with oxygen plasma (O_2 pressure:0.2 mbar, 100 W power) (Fig. S3). During the treatment process, a thin glass-like layer with a stiffness exceeding that of the elastomeric substrate by several orders of magnitude is formed.

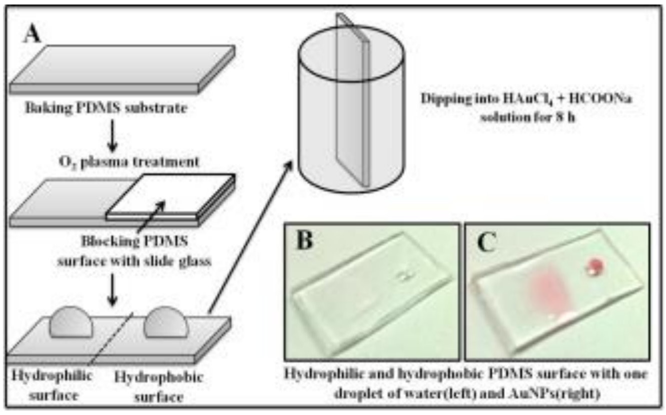

Figure S3: PDMS substrate preparation and its surface modification.

Ultra Violet-visible Spectra of film

The absorbance spectra of the bulk solution and film showed the absence of plasmonic peaks in the remaining solution after the Au NPs were deposited onto the hydrophobic PDMS surface (Fig. S4).

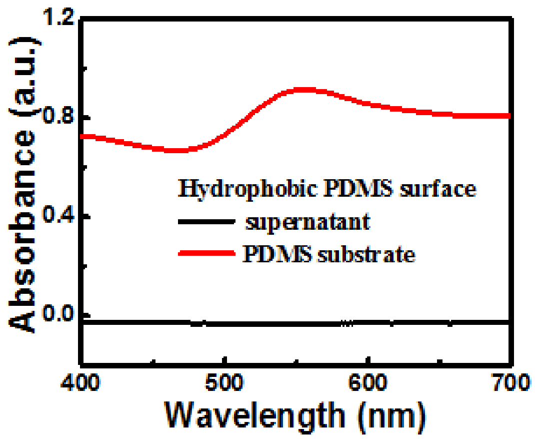

Figure S4: UV-visible spectra of Au NPs film deposited PDMS substrate and supernatant solution.

Morphological Study of Nanostructured films

The morphology of the Au films deposited onto the substrates was observed using SEM (Fig. S5). In the PDMS substrates, a remarkable difference was observed in the Au film deposited onto the as-prepared and plasma-treated PDMS surfaces. The density of the Au NPs on the plasma-treated PDMS was much lower than that of the as-prepared PDMS. However, no difference was observed between the as-prepared and patterned PDMS surfaces. A similar phenomenon was observed for the glass substrates, where the density of Au NPs on the hydrophobic (OTS treated) and relatively hydrophobic (untreated) surfaces was much higher than that on the hydrophilic surface (piranha treated).

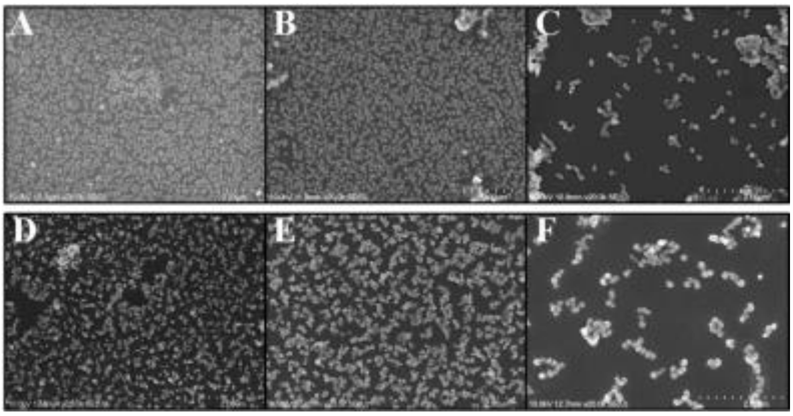

Figure S5: SEM images of the nanostructured films. SEM images of the Au NP films on PDMS (A–C) and glass (D–F) surfaces with different treatments.

Characterization of CdTe QDs and (+)Au NPs

The UV and photoluminescence (PL) spectra were recorded using a Tecan infinite M 200 spectrophotometer (Tokyo, Japan). The samples were excited at 380 nm, and the exciting slit and the emission slit were 5 and 10 nm, respectively. The absorbance and PL intensity of QDs are shown in Figure S6A.The absorbance shoulder of QDs is located at 502 nm, while the PL peak of QDs is situated at 522 nm. According to Peng's equation[2], the particles size of QDs was estimated about 2.85 nm and its concentration was 2.17×10^{-6}M.

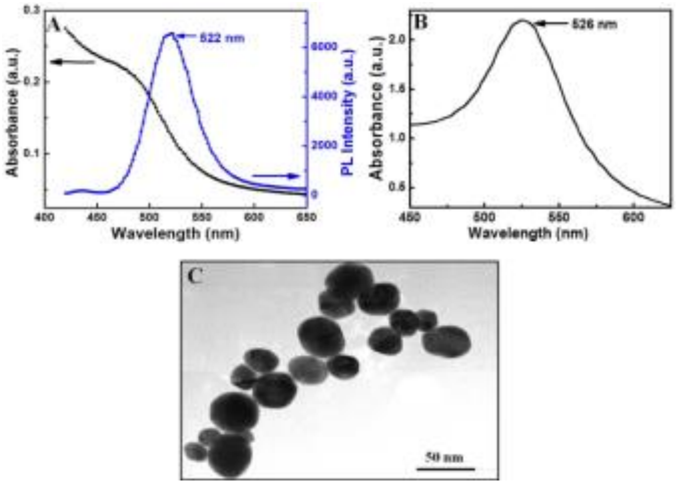

Figure S6: (A) UV and PL spectra of CdTe QDs;(B) UV spectra of positively charged Au NPs and (C) Transmission electron microcopy image of (+)Au NPs.

The absorbance spectra of the synthesized (+)Au NPs are shown in Figure S6B. The absorbance peak of the (+)Au NP solution was located at 526 nm and concentration calculated based on Haiss's method[3]was 3.5×10^{-10} M. The surface charge of (+)Au NPs was +34.5 mV (Zetasizer, Nano-ZS, Malvern, UK). Furthermore, transmission electron microscope revealed (+)Au NP with an average size of 40 nm (Fig. S6C).

AFM image of Au NPs deposited on wrinkled PDMS substrate

In wrinkled PDMS substrate, it's very hard to deposit Au NPs spontaneously on down part of the substrate due to narrow gap between two tip position (2 μm) (Fig. S7). Therefore, a huge amount of Au NPs deposited on tip position of PDMS substrates in compare to down position.

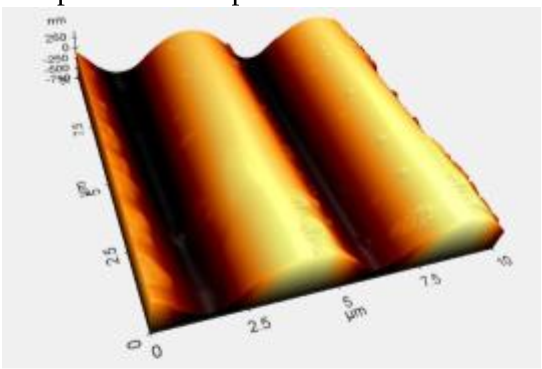

Figure S7: AFM image of Au NPs film on wrinkled PDMS substrate.

Stability and confirmation of adlayer functional group on film

Before do bioassay experiment, the presence of adsorbed adlayer formic acid (HCOOH) on film and its stability in water and under UV light was examined. As shown in Figure S8, FTIR peaks at 1350 cm^{-1}, 1625 cm^{-1} and 3000-25000 cm^{-1} represents the presence of symmetric OCO stretch, asymmetric OCO stretch and carboxylic group O-H stretch on Au NPs films. Zeta potential value of Au NPs film was measured at -36.5 eV. In addition, adlayer functional group showed strong stability under UV light irradiation and water with no change of characteristic functional group in FTIR spectra.

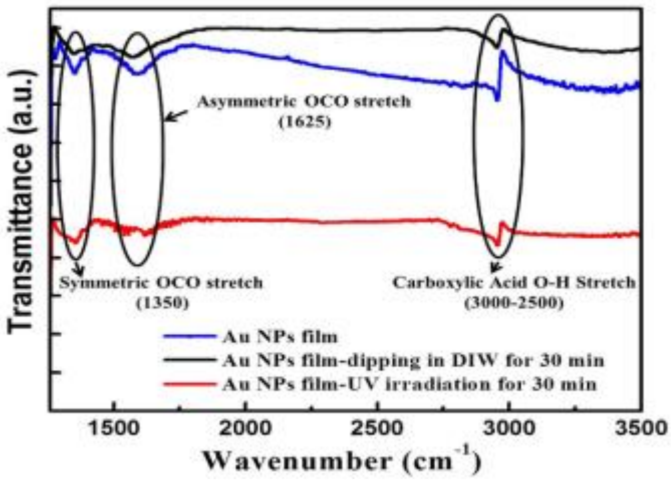

Figure S8: FTIR spectra of Au NPs films under different conditions.

Antibody binding check on film

To illustrate the feasibility of using Au nanostructured films for bioassay applications, antibodies against influenza virus HA were conjugated with the Au NP films deposited onto a 96-well polystyrene plate. The ELISA results confirmed the successful conjugation of the anti-influenza A virus HA H1 antibodies (HA Ab 66189, Abcam, Cambridge, UK) with the Au NP films. As shown in Fig. S9, both the layer-by-layer and EDC/NHS chemistry methods resulted in a higher optical density of the anti-HA antibody-conjugated Au NP films than that of the unmodified Au NP films, suggesting that the nanostructured film was successfully conjugated to the antibodies.

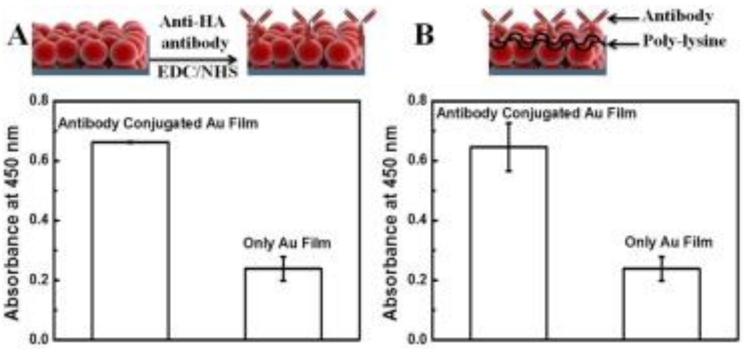

Figure S9: ELISA test of antibody binding to the film. Binding of the anti-HA (H1N1) Ab 66189 antibody to the nanostructured Au films using (A) EDC/NHS chemistry and (B) the layer-by-layer method (right).

Detection of avian influenza virus

The versatility of proposed sensing method was also tested on glass substrate fabricated with Au NPs film using avian influenza virus. After confirmed successful conjugation of anti-HA antibodies with Au NPs film and anti-NA antibodies with (+) Au NPs (Fig. S10A), different concentrated recombinant avian influenza virus (A/Avian/Vietnam 1203/04, H5N1) was mixed with those bio-functionalised nanomaterials. A linear response up to 10 ng mL^{-1} with LOD value of 4.5 ng mL^{-1} was successfully observed and proved the versatility of proposed sensing method (Fig. S10A).

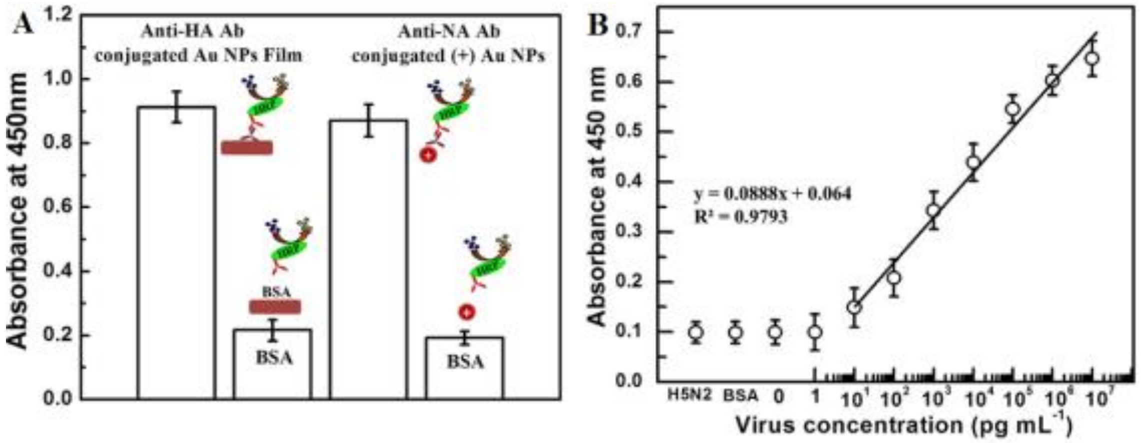

Figure S10: ELISA test of antibody binding and sensing results. (A) Binding of the anti-HA (H5N1) Ab 135382 antibody and anti-NA (H5N1) antibody to the nanostructured Au films and (+) Au NPs respectively. (B) The calibration curve of the absorbance corresponding to the concentration of the influenza virus A/Avian/Vietnam/1203/04 (H5N1). BSA was used as a negative control; H5N2 was used to check specificity of the system. Error bars in (A) and (B) denote standard deviation (n=3).

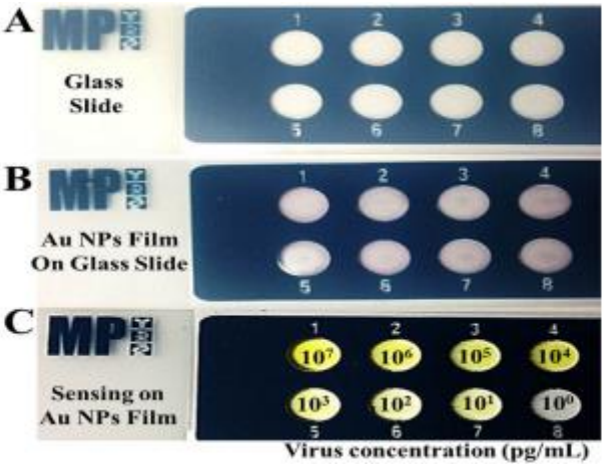

Figure S11: A photographic image of sensing on glass slide. (A) Cleaned glass slide; (B) Au NPs fabricated on glass slide and (C) color developments on glass slide during sensing experiment.

Reference

1. Hanske, *et al.* Strongly coupled plasmonic modes on macroscopic areas via template-assisted colloidal self-assembly. *Nano Lett* **14**, 6863–6871 (2014).
2. Yu *et al.* Experimental Determination of the Extinction Coefficient of CdTe, CdSe, and CdS Nanocrystals. *Chem Mater* **15**, 2854–2860 (2003).
3. Haiss, *et al.* Determination of size and concentration of gold nanoparticles from UV-vis spectra. *Anal Chem* **79**, 4215–5221 (2007).
